# Liquid Biopsy in Hepatocellular Carcinoma: The Significance of Circulating Tumor Cells in Diagnosis, Prognosis, and Treatment Monitoring

**DOI:** 10.3390/ijms241310644

**Published:** 2023-06-26

**Authors:** Mohammed Rifat Shaik, Prem Raj Sagar, Nishat Anjum Shaik, Navkiran Randhawa

**Affiliations:** 1 Department of Medicine, University of Maryland Medical Center Midtown Campus, Baltimore, MD 21201, USA; 2 Franciscan Health Olympia Fields, Olympia Fields, IL 60461, USA

**Keywords:** liquid biopsy, circulating tumor cells, hepatocellular carcinoma

## Abstract

Hepatocellular carcinoma (HCC) is an aggressive malignancy with poor outcomes when diagnosed at an advanced stage. Current curative treatments are most effective in early-stage HCC, highlighting the importance of early diagnosis and intervention. However, existing diagnostic methods, such as radiological imaging, alpha-fetoprotein (AFP) testing, and biopsy, have limitations that hinder early diagnosis. AFP elevation is absent in a significant portion of tumors, and imaging may have low sensitivity for smaller tumors or in the presence of cirrhosis. Additionally, as our understanding of the molecular pathogenesis of HCC grows, there is an increasing need for molecular information about the tumors. Biopsy, although informative, is invasive and may not always be feasible depending on tumor location. In this context, liquid biopsy technology has emerged as a promising approach for early diagnosis, enabling molecular characterization and genetic profiling of tumors. This technique involves analyzing circulating tumor cells (CTCs), circulating tumor DNA (ctDNA), or tumor-derived exosomes. CTCs are cancer cells shed from the primary tumor or metastatic sites and circulate in the bloodstream. Their presence not only allows for early detection but also provides insights into tumor metastasis and recurrence. By detecting CTCs in peripheral blood, real-time tumor-related information at the DNA, RNA, and protein levels can be obtained. This article provides an overview of CTCs and explores their clinical significance for early detection, prognosis, treatment selection, and monitoring treatment response in HCC, citing relevant literature.

## 1. Introduction

Hepatocellular carcinoma (HCC) ranks as the sixth most commonly diagnosed cancer globally. It is now the third leading cause of cancer-related mortality in the general population and also the leading cause of mortality among patients with cirrhosis [1,2]. Its increasing incidence is primarily attributed to the widespread hepatitis C epidemic and the rising prevalence of nonalcoholic fatty liver disease (NAFLD) [3,4]. Other significant risk factors for HCC include chronic liver disease or cirrhosis resulting from hepatitis B virus infection, alcohol-related liver disease, and, less commonly, hemochromatosis, primary biliary cholangitis, and α1-antitrypsin deficiency [5,6,7].

The definitive therapies are surgical resection and liver transplantation (LT), which are only feasible for patients in the very early (0) and early (A) stages [8]. However, percutaneous ablative therapies such as radiofrequency ablation (RFA) and microwave ablation (MWA) have emerged as the preferred initial treatment options for these stages due to their comparable survival benefits, less invasiveness, and lower costs compared to surgical resection [9]. Despite advancements in screening and surveillance programs, a significant majority of the cases (65–70%) are still diagnosed at the intermediate (stage B) or advanced (stage C) stages, making patients ineligible for curative therapies [10]. Consequently, “non-curative” or “palliative” transarterial and systemic therapies are considered and are associated with lower 5-year survival rates [11,12]. Therefore, early diagnosis is paramount in improving survival rates.

Commonly used methods for surveillance include abdominal ultrasonography (US) and/or elevated serum α-fetoprotein (AFP) levels (>20 ng/mL) [13]. However, AFP is suboptimal for early detection, as it can be elevated in non-HCC conditions, and its sensitivity for early-stage tumors is low (10–20%) [14,15]. About 40% of the HCCs do not exhibit elevated AFP levels [16]. Combining US with AFP testing can improve the sensitivity of early detection from 45% to 63% [17]. Although abdominal US is highly accurate (sensitivity: 58–89%, specificity: >90%), its ability to detect small or early-stage nodules is limited [17,18]. Advanced imaging techniques, such as computed tomography (CT) or magnetic resonance imaging (MRI) with new contrast agents, have revolutionized the diagnosis of early-stage HCC. However, their use for surveillance is not recommended due to their high cost-effectiveness ratio and paucity of data [19,20].

Imaging criteria alone are sufficient for diagnosing HCC, without the need for biopsy confirmation in cirrhotic patients, according to European and American guidelines [21,22]. However, despite the recent advancements in imaging, there are still limitations, particularly in non-cirrhotic patients, those with very small nodules (<1 cm), and nodules that lack the typical imaging features of HCC [18]. In such cases, a liver biopsy remains necessary to confirm the diagnosis. Unfortunately, liver biopsy may not be feasible in a significant proportion (48–55%) of indeterminate lesions due to their small size and location. The false negative rate of liver biopsy can also range from 3% to 11% [23]. Furthermore, the considerable spatial and temporal heterogeneity in HCC highlights the need for more than just a single biopsy to fully comprehend the tumor biology beyond confirming the diagnosis [24].

Therefore, there is an unmet need to discover reliable biomarkers to aid in risk stratification, early detection, prognostication, and assessing response to therapy [21]. This paved the way for the exploration of liquid biopsy as a potential tool for HCC management. This technique involves detecting tumor-related products, such as circulating tumor cells (CTCs), circulating tumor DNA (ctDNA), or tumor-derived exosomes, which are released into the bloodstream or other bodily fluids, like saliva, urine, or cerebrospinal fluid [25,26]. Liquid biopsy offers potential solutions for early diagnosis, predicting prognosis, monitoring disease progression, evaluating treatment outcomes, and detecting disease relapse [27]. Furthermore, it has demonstrated effectiveness in identifying mechanisms of resistance to targeted therapies and may enable the guidance of personalized treatment and precision medicine [28]. Compared to traditional biopsies, liquid biopsy is minimally invasive, relatively faster, more cost-effective, and allows for deciphering tumor heterogeneity, which is challenging to achieve with conventional biopsy methods [29].

## 2. Circulating Tumor Cells-Definition and Biology

**Figure 1:**
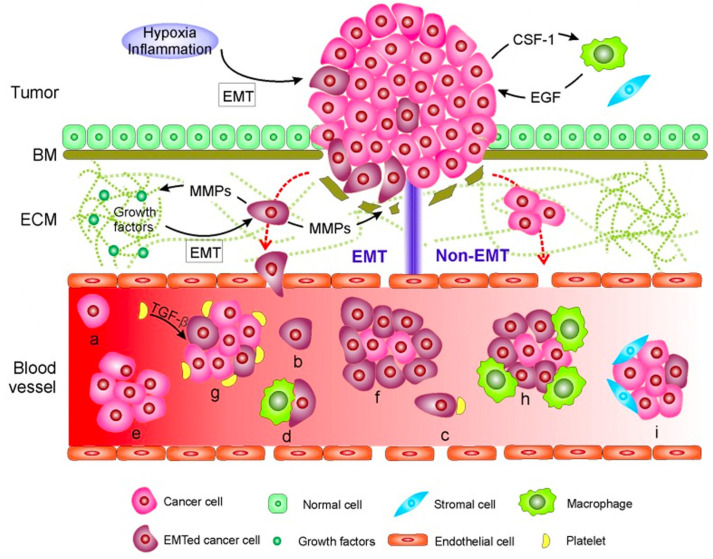
Mechanisms of CTC entry into the bloodstream. The two mechanisms involve EMT-mediated or non-EMT-mediated invasion. In EMT-mediated invasion, tumor cells undergo changes that facilitate the breakdown of the basement membrane (BM) and extracellular matrix (ECM). Non-EMT-mediated invasion is centrosome amplification-triggered or passive infiltration from external forces. MMP: Matrix Metalloproteinases, EGF: Epidermal Growth Factor. (a) Cancer cell; (b) Cancer cell that had undergone EMT; (c,d) Single cancer cells that bind platelets (c) or macrophages (d); (e–i) Cancer cells seen in a cluster with other cancer cells (e,f), platelets (g), macrophages (h) or stromal cells (i) [34].

CTCs were first observed in 1869 during an autopsy of a woman with metastatic breast cancer [30]. These are shed from the primary or metastatic tumor into the bloodstream. The mode of entry into the bloodstream can be via active or passive mechanisms (Figure 1). Passive entry occurs when external forces, such as tumor growth, displace tumor cells [31]. Tumors often induce the formation of new blood vessels (angiogenesis) via the secretion of vascular endothelial growth factor (VEGF) [32]. As the tumor grows and exerts outward pressure, individual cancer cells or clusters of cells (micro emboli) can be forced through these leaky vessels into the bloodstream [33]. Such cells are more likely to retain their original phenotype and may express markers such as EpCAM (epithelial cell adhesion molecule) [33].

Active entry involves various mechanisms, including epithelial-to-mesenchymal transition (EMT) and non-EMT-mediated translocation [35]. In EMT, tumor cells undergo a series of changes that enable them to detach from the primary tumor and acquire characteristics of mesenchymal cells, which are more migratory and invasive [36]. Hypoxia and paracrine signaling from stromal cells can activate transcription factors (e.g., SNAIL, TWIST, and ZEB), microRNAs, and other regulatory elements, leading to EMT [37,38,39]. This results in a loss of tight and adherens junctions between cells, cytoskeletal changes, downregulation of epithelial markers (EpCAM and E-cadherin), and upregulation of mesenchymal markers. Upregulation of enzymes such as matrix-metalloproteinases (MMPs) and cathepsins facilitates tumor cell migration through the stroma and into the bloodstream [40].

Non-EMT-mediated translocation occurs independent of EMT and involves the loss of cell-to-cell adhesion [33]. For example, centrosome amplification can disrupt cell–cell adhesion via increased Arp2/3-dependent actin polymerization, as demonstrated by Godinho et al. [41].

CTCs are predominantly epithelial at the tumor efferent vessels but may switch to a mesenchymal phenotype via Smad2 and β-catenin-mediated signaling pathways [42]. They disseminate through the portal venous and systemic circulations [43]. They undergo a dynamic process of aggregation and disaggregation as well as changes in shape and size in the bloodstream [44]. The lifespan is relatively short, lasting from 1 to 2.4 h [44]. However, some can persist for longer periods due to additional functional gains, such as resistance to anoikis and evasion of the immune system [35]. Some can also intravasate into distant organs and establish a supportive environment in local tissues [45].

CTCs stand out from other liquid biopsy markers because they are a definitive indication of viable tumors, even when conventional imaging methods fail to detect them [46]. Their diagnostic value in early-stage HCC remains a topic of debate [47]. However, they have prognostic value and can serve as markers of treatment response. High numbers of CTCs are associated with poor clinicopathological characteristics, including tumor spread, metastasis, and recurrence. Monitoring changes in CTC counts over time can provide valuable insights into treatment efficacy and disease progression [48,49]. Furthermore, CTCs offer a wealth of information about the molecular characteristics of tumors, including abnormal protein expression, genomic mutations, and mRNA variations. This molecular profiling can shed light on the mechanisms of tumorigenesis, metastasis, and drug resistance, providing valuable insights for personalized treatment strategies [17]. The analysis of molecular alterations through CTCs has the potential to become a non-invasive diagnostic approach, especially for combined hepatocellular-cholangiocarcinoma (cHCC-CCA), and may even replace the need for traditional tissue biopsies [50].

## 3. Techniques of Isolation

CTCs possess unique physicochemical properties, genotype profiles, and cell surface antigens that distinguish them from normal cells. Thus, various immunoaffinity-based, biophysics-based, and enrichment-free techniques can be employed for their isolation (Table 1) [51].

The immunoaffinity technique employs antibodies to target proteins with differential expressions on cells [51]. It can be based on positive or negative enrichment strategies. Negative enrichment involves targeting and removal of background cells, such as leukocytes, to obtain a CTC-enriched sample [52]. For example, the CTC-iChip method depletes white blood cells by targeting CD45, CD16, and CD66b, resulting in a purer CTC population [53]. Negative enrichment approaches offer the advantage of minimal manipulation of CTCs, leading to improved viability, higher recovery rates, and reduced interference [54]. Positive enrichment methods capture CTCs by targeting cell surface markers, such as EpCAM [51].

Immunoaffinity techniques can be further classified into magnetic-based and microfluidic-based devices. The CellSearch assay, which utilizes ferrofluid nanoparticles functionalized with an EpCAM antibody, is the most commonly used and the only FDA-approved immunomagnetic platform for CTC capture [55]. However, one limitation of EpCAM-based capture is the loss of this surface marker in specific CTC subpopulations, such as those undergoing EMT or representing poorly differentiated and stem-cell-like cells [34]. This has prompted the search for new surface markers. For example, Li et al. utilized a synthetic anti-asialoglycoprotein receptor (ASGPR) antibody for the immunomagnetic separation of HCC CTCs [56]. Microfluidic-based devices rely on nano substrates that provide a larger contact area and allow precise control of fluid flow [52,57]. One example is the CTC-Chip developed by Nagrath et al., which consists of micro-posts functionalized with anti-EpCAM antibodies [58]. Another technology, the CTC-iChip, combines microfluidic and immunomagnetic methods and has demonstrated higher sensitivity for CTC detection compared to the CellSearch assay [59].

Biophysical assays rely on the physical properties of CTCs, including their size, density, electric charge, migratory capacity, and deformability [60]. Microfiltration methods such as the CanPatrol utilize the size difference between CTCs and white blood cells [54,61]. The ISET (Isolation by Size of Tumor cells), a 2D microfiltration system, was employed by Vona et al. to detect CTCs in HCC patients undergoing liver resection [62]. However, these techniques may result in the loss of CTCs that are similar in size or smaller than the pore diameter of the capturing device. Additionally, larger molecules and leukocytes can be inadvertently captured. Despite these limitations, the ease of use, high-throughput nature, and good recovery efficacy of microfiltration methods contribute to their continued use [34]. Other biophysics-based platforms, such as those utilizing differential inertial focusing, dielectrophoresis, or photoacoustic resonance effects, have also been developed, although less commonly used [63,64].

Enrichment-free platforms isolate CTCs with a little manipulation of cells. Flow cytometry, as demonstrated by Liu et al., is one such platform that utilizes the higher karyoplasmic ratio (HKR) characteristic of CTCs [65]. However, these methods may have lower CTC purity, and the presence of immune cells with similar characteristics may limit its specificity [65]. Additionally, changes in CTC properties, such as EMT, can further complicate the use of these platforms [54,66].

## 4. Clinical Application of Circulating Tumor Cells

**Figure 2:**
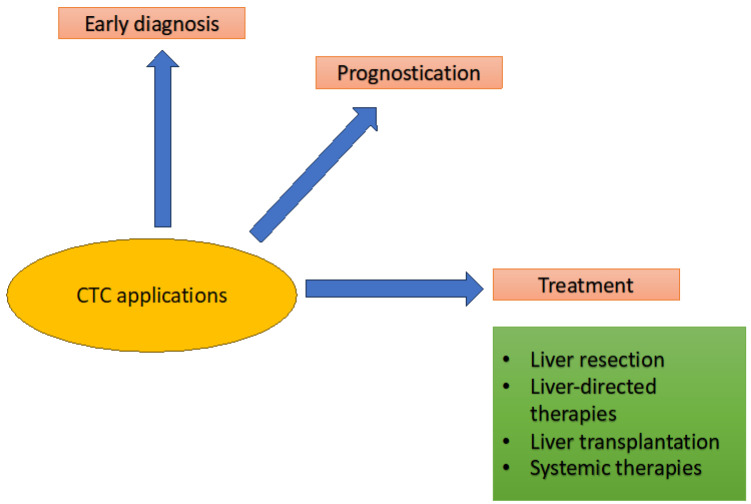
Schematic representation of clinical application of CTCs in the setting of HCC.

As discussed earlier, CTCs have shown promise in various aspects of HCC management, including early diagnosis, prognostication, and monitoring treatment response (Figure 2). These applications are further discussed below.

### 4.1. Circulating Tumor Cells for Early Detection

The use of CTCs as diagnostic markers for HCC has been the subject of several studies. However, the results have been inconsistent, possibly due to the limited expression of certain markers and the varying sensitivity of the isolation method used. For instance, the widely used surface marker, EpCAM positivity, may be present in only 35% of CTCs. Furthermore, there is a low expression in early-stage tumors and loss of expression during EMT [67,68]. To address these issues, researchers have explored the use of liver or HCC-specific markers [such as Glypican-3 (GPC3), ASGPR], mesenchymal markers (Vimentin, Twist, and E-cadherin), and stem cell markers (such as EpCAM, CD133, CD44, CD90, or ICAM-1) [46,69,70,71].

Xu et al. developed a magnetic bead-based system to capture ASGPR+ CTCs, which were then identified using anti-HepPar 1 or anti-CK antibodies via ICC. They discovered CTCs in 81% of HCC patients but later modified the methodology using a new anti-ASGPR monoclonal antibody, resulting in an even higher sensitivity of 89% [56,71]. Chu et al. developed a GPC3-based immunomagnetic fluorescent system (C6/MMSN-GPC3), which improved the capture efficiency by 83.3–350% and isolated CTCs from one early-stage HCC patient, indicating its potential for early diagnosis [72]. Using assays that target multiple surface markers may enhance the detection and isolation of CTCs. For instance, Zhu et al. developed a microfluidic Synergetic-Chip with double antibodies (anti-ASGPR and anti-EpCAM) and achieved a sensitivity of 97.8% and a specificity of 100% at ≥1.5 CTCs/2 mL cutoff [73]. The NanoVelcro assay, which combines EpCAM, ASGPR, and GPC3 antibodies, detected CTCs in 97.6% of patients [74].

The CanPatrol^TM^ system, which employs a positive-enrichment filter-based method and RNA-In Situ Hybridization (RNA-ISH), stratified CTCs into three types: epithelial, mesenchymal, and mixed/hybrid phenotype [48]. Chen et al. used CanPatrol in a cohort of 113 HCC patients and found the total CTC number to be a better diagnostic marker than AFP for HCC detection [75]. Yin et al. also used the CanPatrol technique and found that CTCs positive for Twist were present in 67.5% of HCC patients [76]. Furthermore, Bahsanny et al., by measuring CTCs positive for CK19 and/or CD90 using flow cytometry, could differentiate between chronic hepatitis and HCC with high sensitivity and specificity [77]. Bahn et al. used iChip and IF to isolate liver-specific circulating epithelial cells (CECs) and developed a 25-gene classifier to distinguish between CLD and HCC samples with high sensitivity and specificity [78].

To improve the sensitivity, CTCs can be combined with other biological markers. For example, combining total CTCs and AFP was shown to have even higher sensitivity in diagnosing HCC [75]. Liang et al. found that CTC counts, together with guanine nucleotide-binding protein subunit beta-4 (GNB4) and Riplet gene methylation, can improve early diagnosis with a sensitivity of 88.2% and specificity of 100% [79]. El-Mezayen et al. utilized flow cytometry to identify CTCs (CK18 and CK19) and developed a novel score based on five biochemical blood markers (CK18, CK19, AFP, Albumin, Platelets) to predict HCC among HCV-high-risk patients [80]. Below is a summary of the studies highlighting the utilization of CTCs for HCC detection (Table 2).

Although there have been advances in the utilization of CTCs for diagnosing HCC, a recent meta-analysis of 20 studies found that CTCs have a high probability of error rate, despite their high accuracy [94]. In the early stages of HCC, there are only low levels of CTCs, and the survival rate for those that do enter the bloodstream is even lower [44,95]. Additionally, it is challenging to isolate CTCs from a large number of background cells [95]. The heterogeneity of phenotype and genotype further makes it difficult to develop standardized detection methods [95]. Due to these limitations, CTCs are currently not recommended for HCC surveillance [94].

### 4.2. Circulating Tumor Cells for Prognostication

The presence of CTCs in the peripheral blood is an important indicator of tumor progression, metastasis, and a poor prognosis. Several studies have established a correlation between CTC positivity and/or count with various aspects related to HCC, such as tumor size, portal vein tumor thrombus, AFP levels, degree of differentiation, and disease stage [74,96,97]. Additionally, the presence of CTCs is associated with reduced survival rates. For example, Kelley et al. found that patients with CTCs ≥ 1/7.5 mL were more likely to have AFP ≥ 400 ng/mL (*p* = 0.008) and vascular invasion [87]. Similarly, Sun et al. found that patients with EpCAM-positive CTC counts ≥ 2 had a higher prevalence of satellite foci, vascular invasion, poorly differentiated tumors, and elevated AFP [90]. Schulze et al. demonstrated that EpCAM-positive CTC count (≥1) was associated with vascular invasion, advanced Barcelona Clinic Liver Cancer (BCLC) stage, and elevated AFP [89]. In addition, Liu et al. found that increased numbers of CD45(−) ICAM-1(+) CTCs correlated with reduced disease-free survival (DFS) [88].

Lee et al. established an HCC-CTC mRNA scoring system and found that the HCC-CTC risk score remained an independent predictor of survival after adjustment for MELD (Model for End-Stage Liver Disease) stage, BCLC stage, and CTC count [98]. Chen et al. demonstrated that the presence of clusters of CTCs with immune cells (CTC-WBC) in the bloodstream is an independent predictor of DFS and overall survival (OS) [99].

The mesenchymal phenotype of CTCs (M-CTCs) is more closely associated with tumor aggressiveness [100]. Yang et al. observed a significant association between the presence of M-CTCs and tumor characteristics such as AFP levels ≥ 400 ng/mL, tumor size ≥ 5 cm, multiple tumors, poorly differentiated tumors, incomplete tumor capsule, BCLC stage B or C, microvascular invasion (MVI), and portal vein tumor thrombosis. M-CTC levels were also found to be positively correlated with Ki67 and shorter OS [101]. Table 3 provides an overview of studies investigating the prognostic role of CTCs.

### 4.3. Circulating Tumor Cells in Setting of Treatment

The BCLC staging system is utilized to guide HCC therapy [102]. Treatment options include curative and non-curative interventions. Curative therapies comprise surgical liver resection (LR), orthotopic liver transplantation (OLT), and ablative methods such as thermal ablation. Non-curative treatments include transarterial chemoembolization (TACE), transarterial radioembolization (TARE), and systemic chemotherapy [22].

In early-stage HCC (BCLC stage 0/A), curative therapies are considered. Resection is the preferred treatment for a single tumor < 5 cm without cirrhosis or with cirrhosis but preserved liver function and no significant portal hypertension [103]. Ablation is a cost-effective alternative for early multifocal HCC (two or three nodules smaller than 3 cm) and single small HCCs (<2 cm) without perfectly preserved liver function [9]. OLT is the treatment of choice for early-stage tumors that meet the Milan criteria (single tumor smaller than 5 cm or less than three tumors, each smaller than 3 cm) in the presence of clinically significant portal hypertension and/or decompensated cirrhosis [104].

For BCLC stage B HCC, locoregional TACE therapy is preferred, although TARE has emerged as an alternative [22]. Patients who are ineligible for or experience progression after TACE/TARE should be considered for systemic therapy [22]. For advanced HCC with vascular invasion and/or extrahepatic metastasis (BCLC stage C), the combination of atezolizumab and bevacizumab is now the standard first-line treatment for Child–Pugh A cirrhosis or selected patients with Child–Pugh B cirrhosis [105]. Palliative care is recommended for patients with advanced HCC and Child–Pugh C cirrhosis (BCLC stage D) [106].

CTCs serve as valuable adjuncts to imaging for HCC staging [48]. Measuring CTC counts before and/or after treatment can help predict therapeutic effectiveness and the likelihood of tumor recurrence [107]. Additionally, CTCs can aid in identifying potential resistance to systemic therapies, enabling adjustments in treatment approaches if necessary [46].

#### 4.3.1. In the Setting of Liver Resection

Evidence has shown minimal impact on the CTC count in the immediate postoperative period following surgical resection [108,109]. The decrease in count becomes more apparent within 7–10 days and can persist for up to a month [46]. An increase or persistently high level may be associated with tumor recurrence, extrahepatic metastases, and shorter OS [46].

Several studies have also focused on the predictive value of preoperative CTCs in the setting of curative LR. For instance, the presence of preoperative EpCAM-positive CTCs has been identified as a predictor of recurrence and shorter relapse-free survival (RFS) following LR [110]. Another study discovered that ≥2 preoperative EpCAM-positive CTCs (per 7.5 mL) were associated with an increased likelihood of recurrence, particularly in patients with low AFP levels [90]. Similarly, Hamaoka et al. found that the presence of ≥5 GPC3-positive CTCs was associated with lower DFS and OS rates after LR [70]. Furthermore, Fan et al. found that ≥0.01% levels of cancer stem cells (CSCs) [CD45(−) CD90(+) CD44(+)] in preoperative blood samples can predict intrahepatic recurrence and extrahepatic metastasis [82].

The predictive value of comparing changes in CTC counts pre- and post-surgery for treatment response is still debated [86,111]. While Yu et al. found that patients with increased postoperative CTC counts (from preoperative CTC < 2 to postoperative CTC ≥ 2) had significantly shorter DFS and OS compared to patients with persistent CTC < 2 [109], Xie et al. reported that changes in the CTC number before and after LR did not correlate significantly with postoperative tumor recurrence or metastasis [112].

Interestingly, postoperative CTC counts may have a stronger predictive value than preoperative counts. Zhou et al. found that persistently high numbers of postoperative CTCs (≥5) were associated with an increased risk of early recurrence [108]. Similarly, Sun et al. proposed that a postoperative CTC count of ≥3 could serve as a surrogate marker for predicting extrahepatic metastasis and shorter OS [113].

The mesenchymal phenotype is considered a more robust prognostic indicator, given its enhanced metastatic, invasive, and anti-apoptotic capabilities [112]. Their presence before or after LR is shown to be associated with a higher recurrence rate and worse prognosis [112]. Wang et al. found that having CTCs ≥ 4, mesenchymal CTCs ≥ 1, or mixed CTCs ≥ 3 was positively associated with recurrence [114]. Another study by Qi et al. demonstrated that a preoperative CTC count ≥ 16 and an M-CTC ≥ 2% were significantly associated with early recurrence, multi-intrahepatic recurrence, and lung metastasis [100]. In a separate study by Qi et al., which included 136 HCC patients who underwent complete resection (R0 resection), it was observed that patients with a low CTC count and negative mesenchymal and epithelial/mesenchymal phenotypes had significantly higher tumor-free survival (TFS) rates [115]. Refer to Table 4 for a summary of these studies.

#### 4.3.2. Determining Surgical Margins Prior to Liver Resection

Zhou et al. investigated the relationship between preoperative CTC status and the optimal surgical margin size in HCC patients. They observed that surgical margins > 1 cm were associated with reduced early recurrence rates in the CTC-positive group. Thus, a more extensive surgical margin may be necessary for patients with detectable CTCs to eradicate the disease and minimize the risk of early recurrence. Therefore, with the CTC status before surgery, clinicians are guided about the extent of resection and may achieve better oncological outcomes while preserving liver function [119].

#### 4.3.3. In the Setting of Liver-Directed Therapies

Locoregional liver-directed therapies (LDTs) control the progression of the intrahepatic disease and play a significant role in managing patients who are not surgical candidates. LDTs can also serve as a bridge to LT by maintaining the patient’s eligibility for transplantation. Such therapies include percutaneous ablations and transarterial catheter-directed therapies [120].

MWA has been shown to reduce CTC numbers, whereas RFA and TACE may lead to the release of CTCs [120]. Data have shown that patients who experienced recurrence after MWA had higher levels of serum AFP, AFP-L3 (a specific form of AFP), and CTCs post-treatment compared to their pre-ablation levels. The combination of these markers was found to improve the prediction of recurrence and OS [119].

Wu et al. conducted a retrospective study involving 155 HCC patients who underwent TACE treatment and found that elevated levels of CTCs before surgery were associated with decreased OS, DFS, and 5-year survival rates—a decrease in CTC levels after treatment was associated with positive treatment response [121]. In patients with unresectable HCC who received TACE, Shen et al. demonstrated that the number of EpCAM-positive CTCs was an independent predictor of OS and progression-free survival (PFS) [122].

Thus, monitoring CTC levels before and after LDTs may have prognostic value and provide insights into treatment response and patient outcomes (Table 5).

#### 4.3.4. In the Setting of Liver Transplantation

The Milan criteria are widely utilized to select candidates for LT in the setting of HCC [104]. Various radiological factors and biomarkers have been identified to predict the risk of HCC recurrence after LT; the role of CTCs in predicting such outcomes has been explored in several studies (Table 6) [124].

Chen et al. studied 50 HCC patients and found that pretransplant CTC positivity was associated with early recurrence and poorer prognosis after LT [125]. Similarly, Xue et al. reported that high levels of iFISH-CTCs (>5/7.5 mL) before LT were associated with shorter RFS [91].

The changes in CTC numbers can be erratic after an LT, potentially influenced by immunosuppressive medications. Even a lower count of CTCs in the bloodstream can still pose a risk of tumor recurrence in highly immunosuppressed patients [46]. Postoperative CTC count of ≥1 per 5 mL of blood has been suggested as a useful biomarker to predict post-transplantation recurrence, even in patients who do not meet the traditional Milan, University of California San Francisco (UCSF), or Fudan criteria [126]. Serial CTC detection in the postoperative period may also assist in surveillance for HCC recurrence after surgery [126].

Furthermore, CTCs can help determine eligibility for LT. A prospective study by Court et al. involving 80 HCC patients demonstrated that the presence of vimentin-positive CTCs indicates aggressive underlying disease and occult metastases. These CTCs accurately differentiate early-stage, transplant-eligible patients from transplant-ineligible patients and can predict OS and faster recurrence after curative therapy in early-stage HCC [74].

Thus, the assessment of CTCs before and after LT shows promise in predicting post-transplant outcomes and recurrence risk and guiding patient selection for LT.

#### 4.3.5. In the Setting of Systemic Therapy

Immunotherapy has transformed the treatment landscape for HCC, and liquid biopsy utilizing CTCs has emerged as a potential method for identifying patients likely to benefit from immunotherapy. This has the potential to not only personalize treatment decisions, leading to improved patient outcomes but also reduce healthcare costs by avoiding ineffective treatments and minimizing the risk of adverse events in patients who are unlikely to respond [127].

Nel et al. found variability in the distribution of CTC phenotypes among different patient groups, which can be leveraged to anticipate the effectiveness of therapeutic interventions and identify the most appropriate treatment options. [128]. Li et al. found that a specific CTC phenotype, characterized by ≥40% pERK+/pAkt− CTCs, can serve as a predictive factor for response to sorafenib, a tyrosine kinase inhibitor and was associated with improved PFS [129]. Winograd et al. suggested that the presence of PD-L1+ CTCs may help guide the selection of patients likely to benefit from immune checkpoint inhibitors [130]. Su et al. found that the presence of <2 PD-L1+ CTCs is a positive independent prognostic factor for OS and is associated with a higher objective response rate (ORR) in HCC patients receiving triple therapy [131]. Similarly, Zhang et al. demonstrated the use of a ligand-receptor binding assay on a CTC chip, and Hsieh developed ex vivo culture-based drug sensitivity tests to predict response to chemotherapy [132,133]. These studies are summarized in Table 7.

### 4.4. Clinical Trials Investigating the Use of Circulating Tumor Cells in the Context of Hepatocellular Carcinoma

Various clinical trials (summarized in Table 8) are being conducted in countries, including China, India, Italy, Denmark, and Taiwan, to investigate the role of CTCs in HCC research. Objectives of these trials include evaluating the clinical significance of CTCs in HCC screening and assessing their correlation with OS and DFS in patients undergoing resection. Some aim to explore the association between CTC numbers and tumor characteristics, such as size, number, and BCLC stage. Others focus on analyzing the impact of operative therapies on CTC levels and patient outcomes [134,135,136,137,138,139,140,141,142].

## 5. Challenges and Future Directions

In conclusion, the detection and characterization of CTCs hold great promise for the diagnosis, treatment, and prognosis of HCC. The use of CTCs has the potential to enable personalized treatment strategies. However, several challenges must be addressed before CTCs can be effectively implemented in clinical practice.

The isolation of CTCs is costly, labor-intensive, and time-consuming, requiring large blood sample volumes and sensitive technologies to distinguish these rare cells from millions of other blood cells [143,144]. The multiple steps involved in their isolation can lead to cell apoptosis and reduce cell count, so techniques that improve cell viability and minimize shearing pressures are needed [145]. Furthermore, isolation remains a significant challenge due to their low abundance, even in patients with advanced metastatic disease. Thus, employing CTCs may not be feasible for detecting early-stage HCC [145]. Inconsistent results are also reported due to variations in assay methods and the heterogeneity of CTCs [146]. Standardized protocols are essential to minimize these inconsistencies and ensure reliable results [147]. To increase the effectiveness of testing, CTCs may be combined with other liquid biopsy methods, such as ctDNA and exosomes [148]. Moreover, the current data supporting the utility of CTCs in HCC management mainly comes from proof-of-concept studies, often retrospective and requiring validation via multicenter, prospective trials [149].

These limitations pose challenges in integrating CTC techniques into clinical practice. Replacing existing tools utilized in HCC management with liquid biopsy biomarkers may not be feasible at present. However, there is promising potential for their future integration, which could lead to enhanced predictive capabilities and therapeutic decision-making processes [149,150].

## Figures and Tables

**Table 1:** Techniques for the Isolation of Circulating Tumor Cells [51].

Immunoaffinity	Biophysical	Enrichment-Free Techniques
*Immunomagnetic based techniques*	*Microfiltration based techniques*	ImageStream
CellSearch	Isolation by Size of Tumor cells (ISET)	Photoacoustic flow cytometry (PAFC)
Magnetic-activated cell separation (MACS)	ScreenCell	ELISPOT assay
Surface-enhanced Raman scattering (SERS)	CellSieve	
Subtraction enrichment and immunostaining-fluorescence in situ hybridization (SE-iFISH)	Flexible micro spring array (FMSA)	
	CanPatrol (Microfiltration followed RNA in situ hybridization)	
*Microfluid based techniques*	*Density gradient centrifugation-based techniques*	
CTC-Chip	Ficoll-Paque	
NanoVelcro	OncoQuick	
	RosetteSep CTC Enrichment Cocktail	
*Immunomagnetic as well as Microfluid based techniques*	*Dielectrophoresis based techniques*	
CTC-iChip	DEPArray	

**Table 2:** Diagnostic Role of Circulating Tumor Cells for Hepatocellular Carcinoma.

Scheme	Region	Year of Study	Type of Study	Patients with HCC	Controls	Sensitivity	Technique of Isolation
Armakolas et al. [81]	Greece	2022	Prospective study	89	28 cirrhotic patients	Sensitivity: 46%	qRT-PCR and IF (EPCAM, vimentin, AFP and sMVP)
Bahsanny et al. [77]	Egypt	2014	Prospective study	120	30 with chronic hepatitis C, 33 healthy controls	CK19(+) CTCs: 87.1%/82.5%	Flow cytometry (CK19, CD133 and CD90) and RT-PCR
						CD90(+) CTCs: 82.5%/89.6%	
						CD133(+) CTCs: 40.0%/6.3%	
Bahn et al. [78]	USA	2018	Prospective study	54	39 with chronic liver disease, 10 healthy controls	81% (CTC ≥ 5/10 mL)	CTC-iChip followed by IF staining for glypican-3
Cheng et al. [75]	China	2015–2017	Prospective study	113	57 with chronic liver disease	Total CTCs ≥ 3: sensitivity 62%, specificity 90%, Epithelial CTCs ≥ 1: sensitivity: 45 %, specificity: 79%	CanPatrol
						Mixed CTCs ≥ 2: sensitivity: 53.1%, specificity: 82.5%, Mesenchymal CTCs ≥ 1: sensitivity: 49.6%, specificity: 87.7%	
Chu et al. [72]	China	2021	Prospective study	20	3 healthy volunteers	Cell recovery increased from 42% to 80.3% compared with MACS^®^ Beads	Glypican-3 (GPC3)-based immunomagnetic fluorescent system
Fan et al. [82]	China	2005–2009	Prospective study	82	-	Sensitivity: 68.3%	Multicolor flow cytometry—CSCs (CD45 − CD90 + CD44+)
Fang et al. [83]	China	2012–2013	Prospective study	42	-	CTCs (≥1/5 mL): 74%/100%	CellSearch
Guo et al. [84]	China	2006	Prospective study	44	7 healthy controls	AFP mRNA (sensitivity, specificity, diagnostic accuracy): 50%, 76.5%, 86.7%	RT-PCR followed by CD45 and Ber-EP4 immunomagnetic beads
Guo et al. [85]	China	2012–2013	Prospective study	299	71 healthy donors, 24 with benign tumor, and 25 with chronic hepatitis B and/or liver cirrhosis	EpCAM-mRNA (+) CTCs (sensitivity, specificity): 42.6%/96.7%	CellSearch and qRT-PCR
Guo et al. [86]	China	2012–2015	Multicenter Clinical Trial	395	201 with chronic hepatitis B and/or liver cirrhosis, 100 with benign liver lesions, 210 healthy controls	Sensitivity/specificity: 72.5%/95%	Multimarker qRT-RNA detection platform
Kalinch et al. [52]	USA	2017	Prospective study	63	26 with chronic liver disease, 34 healthy donors	Out of 15 patients who were tested for both AFP and CTC scores, 33% were detected by both assays, 27% were detected by CTC score alone, and 7% were detected by AFP alone. Either the AFP or CTC score was positive in 67%	CTC-iChip RNA-based digital qRT-PCR
Kelley et al. [87]	USA	2011–2012	Prospective study	20	10 with non-malignant liver disease	1 CTC/7.5 mL	CellSearch
						AFP ≥ 400 ng/mL: sensitivity 70%, AFP < 400 ng/mL:	
						Sensitivity: 10%	
Li et al. [56]	China	2013	Prospective study	27	34 with benign liver disease/hepatitis/cirrhosis and 15 healthy volunteers.	Sensitivity: 89%	anti-ASGPR, CPS1 and P-CK antibodies
Liang et al. [79]	China	2020–2022	Prospective study	17	11 cases of HBV-related decompensated cirrhosis	70.6%/90.9%	CTCBIOPSY device
Liu et al. [88]	China	2013	Prospective study	60	--	High CD45-ICAM-1+ cell frequency (>0.157%)–50%	CD45-ICAM-1+
Schulze et al. [89]	Germany	2013	Prospective study	59	19 with cirrhosis or benign hepatic tumor	Sensitivity: 30.5%	CellSearch
Sun et al. [90]	China	2010–2011	Prospective study	123	20 healthy volunteers	CTC ≥ 2: 71%/80%	CellSearch
Xu et al. [71]	China	2009	Prospective study	85	37 with benign liver diseases	CTC positivity: 81%	ASGPR (+)
Xue et al. [91]	China	2014–2015	Prospective study	30	10 healthy volunteers	Cellsearch-CTCs: 27%/100%; iFISH-CTCs: 70%/100%	CellSearch and iFISH (CK+/DAPI+/CD45−)
Yao et al. [92]	China	2003–2004	Prospective study	49	18 healthy donors, 16 with cirrhosis, 20 with hepatitis	Sensitivity: 72.1%	CD45 and Ber-EP4 immunomagnetic beads followed by AFP mRNA-nested RT-PCR
Yin et al. [76]	China	2015–2017	Prospective study	80	10 healthy volunteers	Sensitivity: 77.5%	CanPatrol
Zhou et al. [93]	China	2012	Prospective study	49	-	CTC ≥ 2: 34.6%/100%	EpCAM mRNA+ CTC detection and qRT-PCR
Zhu et al. [73]	China	2019	Prospective study	45	Six healthy donors and six with benign tumors	≥1.5 CTCs/2 mL: 97.8%/100%	Microfluidic synergetic-chip (anti-ASGPR and anti-EpCAM)

AFP: Alpha-Fetoprotein; EPCAM: Epithelial Cellular Adhesion Molecule; RT-PCR: Reverse Transcription-Polymerase Chain Reaction; IF: Immunofluorescence; sMVP: Surface Major Vault Protein; ASGPR: Asialoglycoprotein Receptor; CPS1: Carbamoyl Phosphate Synthetase 1; P-CK: Pan-cytokeratin; ICAM-1: Intercellular Adhesion Molecule 1; iFISH: Immunofluorescence in Situ Hybridization.

**Table 3:** Prognostic Role of Circulating Tumor Cells for Hepatocellular Carcinoma.

Study	Region	Year of Study	Type of Study	HCC Patient Number	Technique of Isolation	Key Findings
Chen et al. [99]	China	2014–2020	Retrospective analysis	136	CanPatrol, filtration and multiple mRNA ISH	CTC-WBC cluster ≥ 1/5 mL was associated with distant metastasis, tumor relapse and a shorter RFS
Kelly et al. [87]	USA	2011–2012	Prospective study	20	CellSearch	CTCs ≥ 1 per 7.5 mL was associated with AFP ≥ 400 ng/mL and vascular invasion
Liu et al. [88]	China	2013	Prospective study	60	CD45-ICAM-1+	High frequency of CD45-ICAM-1+ cells (≥0.157%) was associated with a shorter DFS and OS. It is an independent risk factor for poor outcomes, including portal vein tumor thrombus and the presence of ascites
Sun et al. [90]	China	2010–2011	Prospective study	123	CellSearch	CTCs ≥ 2 per 7.5 mL was found to be significantly associated with aggressive HCC phenotypes
Schulze et al. [89]	Germany	2013	Prospective study	59	CellSearch	The presence of CTCs was associated with shorter OS advanced BCLC stage (stage C), microscopic vascular invasion, and elevated AFP ≥ 400 ng/mL
Vona et al. [60]	France	2004	Prospective study	44	ISET method	The presence of CTCs was associated with diffuse tumors and portal tumor thrombosis.
Yang et al. [101]	China	2014–2017	Prospective study	105	CanPatrol	M-CTC positivity was associated with AFP ≥ 400 ng/mL, tumor size ≥ 5 cm, the presence of multiple tumors, poorly differentiated tumors, incomplete tumor capsule, BCLC stage B or C, microvascular invasion and portal vein tumor thrombosis

ISH: In Situ Hybridization; RFS: Relapse-Free Survival; TNM: Tumor (T), Nodes (N), and Metastases (M); OS: Overall Survival; DFS: Disease-Free Survival; BCLC: Barcelona Clinic Liver Cancer; ISET: Isolation by Size of Tumor cells.

**Table 4:** Role of Circulating Tumor Cells in the Setting of Liver Resection for Hepatocellular Carcinoma.

Study	Region	Year of Study	Type of Study	HCC Patient Number	Technique of Isolation	Key Findings
Court et al. [74]	USA	2015–2016	Prospective study	61	NanoVelcro assay (ASGPR, Glypican-3, EpCAM)	Vimentin (+) CTCs associated with OS, PFS and portended faster time to recurrence
Fan et al. [82]	China	2005–2009	Prospective study	82	Multicolor flow cytometry—CSCs (CD45 − CD90 + CD44+)	Circulating CSCs > 0.01% predicted: intrahepatic recurrence, extrahepatic recurrence, lower 2-year RFS and OS
Guo et al. [86]	China	2012–2015	Multicenter clinical trial	395	Multimarker qRT-RNA detection platform	Persistently positive CTCs after resection were associated with a higher recurrence rate. CTC load/5 mL > 0.80 was associated with a significantly shorter TTR
Ha et al. [111]	South Korea	2014–2016	Prospective study	105	Tapered slit flter (TSF) platform	Increased CTCs after surgery were associated with a higher level of recurrence. Positive ΔCTC was associated with shorter OS and higher recurrence among patients with low AFP levels and cirrhosis
Hamaoka et al. [70]	Japan	2015–2016	Prospective study	85	Glypican-3(+)	CTCs ≥ 5 was an independent predictor of mPVI and poor prognosis.
Ni et al. [116]	China	2014–2017	Retrospective study	97	CanPatrol, filtration	CTC < 20 and NLR < 2.15 were associated with longer OS. Patients were classified into CTC-NLR (0), CTC-NLR (1), and into CTC-NLR (2). CTC-NLR (0) was associated with the best OS, whereas CTC-NLR (2) had the worst OS
Ogle et al. [117]	UK	2012–2015	Prospective study	69	IF (EpCAM, CK, AFP and GPC3) and size	CTC > 1 per 4 mL blood post treatment was significantly associated with a poorer survival: 7.5 months for >1 CTC versus > 34 months for patients with <1 CTC
Ou et al. [49]	China	2013–2016	Prospective study	165	CanPatrol	Mesenchymal CTCs were associated with high levels of AFP, multiple tumors, advanced TNM and BCLC stage, presence of embolus or micro embolus and the shortest relapse-free survival
Qi et al. [115]	China	2014–2017	Retrospective study	136	CanPatrol	TFS was higher with low CTCs count and M- and E/M-negative phenotypes. High pre-resection CTC count and M- and E/M-positivity associated with extrahepatic and multi-intrahepatic recurrence.
Qi et al. [100]	China	2014–2016	Prospective trial	112	CanPatrol	Post operative CTC count ≥ 16 and M-CTC ≥ 2% were associated with early recurrence, multi-intrahepatic recurrence, and lung metastasis. Postoperative CTC monitoring showed an increase in CTC count and M-CTC % before clinically detectable recurrence nodules appeared.
Sun et al. [90]	China	2010–2011	Prospective study	123	CellSearch	Preoperative CTC (7.5 mL) of ≥2 was an independent prognostic factor for tumor recurrence
Sun et al. [42]	China	2013–2015	Prospective study	73	CellSearch	The presence of CTCs in the hepatic vein, along with the presence of CTM, was an independent prognostic factor for the development of lung metastasis.
Von Felden et al. [110]	Germany	2011–2015	Prospective study	61	CellSearch	CTC-positivity was associated with a higher risk of recurrence and a shorter RFS
Wang et al. [114]	China	2014–2016	Prospective study	62	CanPatrol	Mesenchymal CTC positivity was associated with ER and shortened postoperative disease-free survival
Xie et al. [112]	China	2016–2019	Retrospective study	66	CanPatrol	Recurrence rates of postoperative interstitial CTC-positive and CTC-negative groups: 1-year recurrence: 21.7% vs. 10.8% 2-year recurrence: 37.5% vs. 10.8% 3-year recurrence: 55.5% vs. 10.8%, 1 -, 2- and 3-year recurrence rates of interstitial CTC in the increasing group were 25.2%, 36.9% and 66.9% 1-year recurrence: 21.7% vs. 10.8%
Ye et al. [118]	China	2014–2017	Prospective study	42	CanPatrol	Postoperative CTC counts (≥2 and ≥5) and pre/postoperative change in CTC counts were significantly associated with PFS
Yu et al. [109]	China	2013–2015	Prospective study	139	CellSearch	Increase in postoperative CTC counts (from preoperative CTC < 2 to postoperative CTC ≥ 2) is associated with shorter DFS and OS
Zhou et al. [93]	China	2012	Prospective study	49	EpCAM mRNA (+)	Post-operative CTC ≥ 2.22 was an independent prognostic biomarker for early recurrence

CTM: Circulating Tumor Microemboli; mPVI: macroscopic Portal Vein Invasion; TTR: Time-To-Recurrence; ΔCTC: Change in CTC count; DFS: Disease Free Survival; ER: Early Recurrence; TFS: Tumor-Free Survival; NLR: Neutrophil-Lymphocyte Ratio; CSC: Cancer Stem Cells; M-CTC: Mesenchymal CTC; PFS: Progression-Free Survival.

**Table 5:** Role of Circulating Tumor Cells in the Setting of Liver Directed Therapies for Hepatocellular Carcinoma.

Study	Region	Year of Study	Type of Study	HCC Patient Number	Technique of Isolation	Key Findings
**Trans arterial Chemoembolization (TACE)**						
Chen et al. [123]	China	2017–2018	Retrospective analysis	107; treated with TACE and MWA	Cyttel method	Pretreatment CTC count and EMT phenotypes were not predictive of short-term efficacy. Comprehensive therapy reduced the total CTC and mesenchymal CTC count
Guo et al. [85]	China	2012–2013	Prospective study	299 HCC; 157-curative resection, 76-TACE, and 66-radio therapy	EpCAM (mRNA+)	Pretreatment CTC level showed prognostic significance in patients treated with resection, TACE, and radiotherapy. Preoperative detectable EpCAM mRNA+ CTCs had significantly shorter TTR and higher recurrence rates. A decrease in CTC levels after treatment reflected tumor response. Persistent positive CTCs (preoperative and postoperative) was associated with higher recurrence rates.
Shen et al. [122]	China	2014–2015	Prospective study	89	CellSearch	Pretreatment CTC counts were independent predictors of OS and PFS.
Wu et al. [121]	China	2012–2014	Retrospective analysis	155	Immunomagnetic beads and FISH (chromosome 8 amplification)	Positive preoperative CTCs were associated with lower OS, DFS, and 5-year survival rates
**Microwave ablation (MWA)**						
Zhou et al. [119]	China	2014–2017	Prospective study	105	CellSearch	Combined detection of serum AFP, AFP-L3, CTCs improves the prediction of recurrence after MWA

CTM: Circulating Tumor Microemboli; mPVI: macroscopic Portal Vein Invasion; TTR: Time-To-Recurrence; ΔCTC: Change in CTC count; OS: Overall Survival; DFS: Disease Free Survival; ER: Early Recurrence; TFS: Tumor-Free Survival; NLR: Neutrophil-Lymphocyte Ratio; CSC: Cancer Stem Cells; M-CTC: Mesenchymal CTC; PFS: Progression-Free Survival.

**Table 6:** Role of Circulating Tumor Cells in the Setting of Liver Transplantation (LT) for Hepatocellular Carcinoma.

Study	Region	Year of Study	Type of Study	HCC Patient Number	Technique of Isolation	Key Findings
Chen et al. [125]	China	2016–2019	Retrospective study	50	Negative enrichment (anti-CD45) and iFISH	CTCs positivity correlated with tumor size, AFP level, tumor grade and recurrence. CTC-negative vs. CTC-positive: 1-year DFS: 91.6% vs. 61.5% (*p* = 0.02), 1-year OS: 88.5% s. 91.7% (*p* = 0.75)
Court et al. [74]	USA	2015–2016	Prospective study	61	NanoVelcro assay (ASGPR, Glypican-3, EpCAM)	Vimentin (+) CTCs accurately discriminated early-stage, LT eligible patients from locally advanced/metastatic, LT ineligible patients
Wang et al. [126]	China	2017–2019	Prospective study	193	ChimeraX-i120, anti-EpCAM, anti-pan-CK	Post-operative CTC count ≥ 1 per 5 mL predicts recurrence after LT
Xue et al. [91]	China	2014–2015	Prospective study	30	iFISH and CellSearch	iFISH-CTCs < 5/7.5 mL associated with increased RFS

OS: Overall Survival; DFS: Disease Free Survival; RFS: Relapse-Free Survival; iFISH: Immunofluorescence in Situ Hybridization; ASGPR: Asialoglycoprotein Receptor; EpCAM: Epithelial Cell Adhesion Molecule.

**Table 7:** Role of Circulating Tumor Cells in the Setting of Systemic Therapies for Hepatocellular Carcinoma.

Study	Region	Year of Study	Type of Study	HCC Patient Number	Technique of Isolation	Key Findings
Li et al. [129]	China	2017	Prospective study	63	CD45- and pAkt1/2/3 or pERK1/2+	≥40% pERK+/pAkt− CTCs showed longer PFS and response to Sorafenib treatment
Su et al. [131]	China	2022	Prospective study	47	CytoSorter	Patients with <2 PD-L1+ CTCs exhibited a higher ORR and longer OS compared to those with ≥2 PD-L1+ CTCs. PD-L1-positive CTCs were an independent predictive biomarker for OS in patients receiving triple therapy.
Winograd et al. [130]	USA	2014–2017	Prospective study	102	NanoVelcro Chip	PD-L1+ CTCs are primarily detected in advanced-stage HCC and independently predict OS when controlling for the MELD score, AFP levels, and tumor stage.

PFS: Progression-Free Survival; OS: Overall Survival; ORR: Objective Response Rate; MELD: Model for End-Stage Liver Disease.

**Table 8:** Ongoing Clinical Trials Involving Circulating Tumor Cells in Setting of Hepatocellular Carcinoma.

Study	Region	Year of Study	Type of Study	HCC Patient Number	Technique of Isolation	Key Findings
NCT04688606 [134]	China	2020–2021	Retrospective study	300	CTCBIOPSY to detect CTC number (including interventional therapy, tumor resection, or LT) 1–3 days before, 1 month after surgery, and 6 months after surgery	To evaluate the clinical significance of CTCs in HCC screening and postoperative recurrence monitoring
NCT05297955 [135]	China	2013–2022	Retrospective study	458	CellSearch to detect CTC number in patients undergoing liver cancer surgery during perioperative period	CTC levels before and after surgery were significantly correlated with OS and DFS. Preoperative CTC correlated with disease-related clinical parameters, while postoperative CTC was an independent prognostic indicator
NCT03162198 [136]	India	2017–2018	Cross sectional study	53	Unclear	To detect CTC number in cirrhotic HCC patients and to correlate CTC number with tumor size, number, and BCLC stage
NCT04521491 [137]	China	2020–2023?	Randomized Controlled Study	184	Unclear	To analyze the effect of postoperative FOLFOX4 therapy after HCC resection based on folate receptor-positive CTCs. Patients were randomized to postoperative FOLFOX4 group and no FOLFOX4 group. The time to recurrence, the OS as well as the incidence of complications after therapy was observed
NCT01930383 [138]	Taiwan	2013–2015	Prospective study	150	Microfluidic disk platform	To explore the correlation between CTC number and clinical characteristics; to compare the patterns of molecular aberrations between CTC and HCC tumor tissue; and to measure the changes in CTCs numbers and molecular aberrations before and after targeted therapy
NCT04800497 [139]	Italy	2019–2024	Prospective study	200	FACSymphony™ and subsequently by EpCAM, N-cadherin and CD90	To evaluate the association between CTCs and DFS/OS
NCT05242237 [140]	China	2021–2024	Prospective study	300	Microfluidic Platform: Cellomics CTC-100 cell sorter	To determine the relationship between the CTC number and prognosis/treatment response, detect mutation, copy number variation and mutation load in CTC cells using single-cell whole genome sequencing technology, and use bioinformatics analysis of CTC heterogeneity and its relationship with clinical outcome
NCT02973204 [141]	Denmark	2016–2020	Prospective study	30	Flow cytometry	Treatment response; To correlate between the CTC number and survival in HCC patients treated with Sorafenib
NCT02727673 [142]	China	2012–2014	Prospective Randomized Trial	500	Unclear	To investigate the relationship between CSCs and postoperative recurrence/metastasis

ISH: In Situ Hybridization; RFS: Relapse-Free Survival; TNM: Tumor (T), Nodes (N), and Metastases (M); OS: Overall Survival; DFS: Disease-Free Survival; BCLC: Barcelona Clinic Liver Cancer; ISET: Isolation by Size of Tumor cells; CSCs: Cancer Stem Cells.

## Data Availability

Data sharing is not applicable. No new data were created or analyzed in this study. Data sharing is not applicable to this article.

## Glossary

AFP	alpha-fetoprotein
Anoikis	programmed cell death upon detachment of cells from the extracellular matrix and neighboring cells
ASGPR	asialoglycoprotein receptor
BCLC	Barcelona clinic liver cancer
C6/MMSN-GPC3	CTC capturing system that utilizes immunomagnetic fluorescent nanodevices targeting GPC3
CECs	circulating epithelial cells
cHCC-CCA	combined hepatocellular-cholangiocarcinoma
CPS1	carbamoyl phosphate synthetase 1
CSCs	: cancer stem cells
CT	computed tomography
CTCs	circulating tumor cells - tumor cells that have detached from the primary tumor and circulate in the bloodstream
CTC-WBC	clusters composed of CTCs and white blood cells circulating in the bloodstream
ctDNA	circulating tumor DNA - tumor-derived fragmented DNA originating from primary or metastatic cancer sites.
DFS	disease-free survival
EHM	extrahepatic metastasis
EMT	epithelial-to-mesenchymal transition – a cellular process in which epithelial cells acquire mesenchymal phenotypes and behavior
EpCAM	epithelial cell adhesion molecule
ER	early recurrence
FISH	fluorescence in situ hybridization
FMSA	flexible micro spring array
GNB4	guanine nucleotide-binding protein subunit beta-4
GPC3	glypican-3
HCC	hepatocellular carcinoma
HKR	higher karyoplasmic ratio
ICAM	intercellular adhesion molecule
ISET	isolation by size of tumor cells
ISH	in situ hybridization
LDT	liver-directed therapy
Liquid biopsy	A laboratory test conducted on a blood, urine, or other body fluid sample to detect cancer cells derived from a tumor or small fragments of DNA, RNA, or other molecules released by tumor cells
LR	liver resection
LT	liver transplantation
MACS	magnetic-activated cell separation
M-CTCs	mesenchymal phenotype of CTCs
MELD	model for end-stage liver disease
MMP	matrix-metalloproteinase
MVI	microvascular invasion
MWA	microwave ablation
MRI	magnetic resonance imaging
mPVI	macroscopic portal vein invasion
NAFLD	nonalcoholic fatty liver disease
NLR	neutrophil-lymphocyte ratio
OLT	orthotopic liver transplantation
ORR	objective response rate
OS	overall survival
PAFC	photoacoustic flow cytometry
P-CK	pan-cytokeratin
PFS	progression-free survival
R0 resection	surgical margin microscopically-negative for residual tumor
RFA	radiofrequency ablation
RFS	relapse-free survival
RT-PCR	reverse transcription-polymerase chain reaction
SE-iFISH	subtraction enrichment and immunostaining-fluorescence in situ hybridization
SERS	surface-enhanced Raman scattering
sMVP	surface major vault protein
TACE	transarterial chemoembolization
TARE	transarterial radioembolization
TFS	tumor-free survival
TTR	time-to-recurrence
Tumor-derived exosomes	small extracellular vesicles secreted by cancer cells
TNM	tumor (T), nodes (N), and metastases
UCSF	University of California San Francisco
US	ultrasonography
VEGF	vascular endothelial growth factor
